# NEXT-peak: a normal-exponential two-peak model for peak-calling in ChIP-seq data

**DOI:** 10.1186/1471-2164-14-349

**Published:** 2013-05-25

**Authors:** Nak-Kyeong Kim, Rasika V Jayatillake, John L Spouge

**Affiliations:** 1 Mathematics and Statistics Department, Old Dominion University, Norfolk, VA 23529, USA; 2 Computational Biology Branch, National Center for Biotechnology Information, National Library of Medicine, National Institutes of Health, Bethesda, MD 20894-6075, USA

**Keywords:** ChIP-seq, Normal-exponential distribution, Continuous mixture, Poisson regression, Goodness-of-fit

## Abstract

**Background:**

Chromatin immunoprecipitation followed by high-throughput sequencing (ChIP-seq) can locate transcription factor binding sites on genomic scale. Although many models and programs are available to call peaks, none has dominated its competition in comparison studies.

**Results:**

We propose a rigorous statistical model, the normal-exponential two-peak (NEXT-peak) model, which parallels the physical processes generating the empirical data, and which can naturally incorporate mappability information. The model therefore estimates total strength of binding (even if some binding locations do not map uniquely into a reference genome, effectively censoring them); it also assigns an error to an estimated binding location. The comparison study with existing programs on real ChIP-seq datasets (STAT1, NRSF, and ZNF143) demonstrates that the NEXT-peak model performs well both in calling peaks and locating them. The model also provides a goodness-of-fit test, to screen out spurious peaks and to infer multiple binding events in a region.

**Conclusions:**

The NEXT-peak program calls peaks on any test dataset about as accurately as any other, but provides unusual accuracy in the estimated location of the peaks it calls. NEXT-peak is based on rigorous statistics, so its model also provides a principled foundation for a more elaborate statistical analysis of ChIP-seq data.

## Background

ChIP-seq experiments use chromatin immunoprecipitation and then high-throughput sequencing, primarily to locate transcription factor binding sites across entire genomes, and to better our understanding of biological control systems [1]. As a brief overview of the relevant experimental protocols, they begin by irreversibly cross-linking a transcription factor (TF) molecule to its binding site in genomic DNA. They then shear the DNA into millions of short sequence fragments. Usually, the ends of a fragment are near the corresponding cross-link, but the exact distance between the end of the fragment and the cross-link is random. Moreover, the fragments on the two DNA strands show different systematic biases in the positions of their ends relative to the cross-link. Antibodies to the TF then precipitate each TF molecule along with its attached fragment. Fragments are dissociated from the TF molecules, amplified by polymerase chain reaction (PCR). The fragments are then sequenced into short subsequences, called “tags”. Computational analysis then enters by mapping the tag sequences to a reference genome. If a tag sequence is long enough, the tag matches only one genomic coordinate. Sometimes, however, its sequence is short and maps to more than one coordinate, making the mapping ambiguous. The possibilities of ambiguous mapping, false positive tag-reads, and other experimental errors have motivated the development of programs to analyze ChIP-seq experiments.

Peak-calling programs locate potential binding sites as “peaks” where mapped tags concentrate. Peak-calling programs use widely differing approaches, none of which has yet emerged as dominant in reviews [2-4], because relative accuracy of programs varies with the dataset examined [2,3]. Improvement is probably possible, however, because the models underlying existing programs do not consider mapping ambiguities directly, despite the existence of packages for enumerating the ambiguities, e.g., the PeakSeq suite [5]. Moreover, some programs ignore strand-specific information [6,7].

Most programs use (sometimes implicitly) kernel smoothing to compensate for mapping ambiguities, the most popular kernel being the uniform density, which is equivalent to counting tags in sliding windows of fixed-length [8-12]. Programs also manipulate information about a tag’s strand in various ways: as mentioned, some ignore it [6,7]; some use it explicitly (e.g. SISSRs [12], spp [11]); and some use it indirectly by transposing tag locations over to the other strand (e.g. MACS [8], QuEST [13], CisGenome [9]). Programs that combine strand information and windowing are essentially using two separate uniform densities as kernels for the forward and backward strands. For example, QuEST [13] (among other computer programs) estimates tag densities with a kernel smoother than the uniform density, to mimic the observed shape of tag peaks. QuEST did not dominate in comparisons, however, perhaps because it transposes tag locations, rather than estimating two separate tag densities, one for each strand.

The significance of peaks can be reported either as the number of tags in a window, a p-value, a q-value, or a posterior probability. Although p-values guide a naïve user better than tag numbers, they introduce problematic assumptions. To derive a p-value, some programs [7,13] assume a globally uniform background intensity of tags, an assumption known to fail in ChIP experiments. To assign the significance of peaks, different programs use various model assumptions such as Poisson [7,12,13], local Poisson [8], binomial [9], and hidden Markov [10] models. Some programs [8,9,11-13] use control data to account for local variations in background tag intensity or to compensate for experimental artifacts like PCR over-amplification, which can cause a spurious concentration of tags in a few specific locations. The reproducibility of control data is suspect, however, because it varies across cell types and ChIP protocol [4]. Although control data mitigates some experimental artifacts, its unreliability ultimately undermines any inference based on the corresponding p-value.

Mapping ambiguities can also be problematic for a naïve p-value calculation. Consider, e.g., a large genomic region where exactly *A* locations are ambiguously mapped (where *A* is fixed). In the same region, consider a window containing a total of *L* locations, including the *A* ambiguous locations. The window length is an arbitrary parameter (within limits), and as it increases, *L* increases. The *A* ambiguous locations are essentially censored data, so the simplest maximum likelihood estimate of the tag count in the window is *L*/(*L-A*) times the observed tag count. Thus, if observed tag count is fixed, the estimated tag count decreases as the window length increases. If a p-value decreases with the estimated tag count, it then depends on the window length. False discovery rates (FDR) depend on p-values, so the use of FDRs does not remove the dependency. Under the circumstances described, therefore, the arbitrary choice of window length influences the number of peaks reported. A recent study on the number of binding sites in a genome [14] indicates that many real binding sites from ChIP-seq data go unreported, suggesting that the assumptions and approximations underlying current p-value estimates leave room for improvement.

Intuitively, the spatial resolution of a peak should also improve as more tags contribute to it. In principle, therefore, a program should also assign errors to its location estimates, but in fact, existing programs do not infer the accuracy of their estimated peak locations.

To examine the performance of the proposed model (NEXT-peak) against current standards, we selected several programs from a recent comparison [2]: HPeak [10], spp package (WTD and MTC) [11], CisGenome [9], MACS [8], QuEST [13], and SISSRs [12]. A summary of selected programs is given in Table 1. The details of NEXT-peak appears in the Methods.

**Table 1:** Summary of programs used for comparison

	**Profile**	**Strand specific**	**Statistical model**	**Peak criteria**	**Rank by**	**Ref**
HPeak	Sliding window	Indirect	Hidden Markov model	Tag counts	Tag counts	[10]
WTD	Sliding window	Direct		Score	P-value	[11]
MTC	Sliding window	Direct		Score	P-value	[11]
CisGenome	Sliding window	Indirect	Binomial	Tag counts	Tag counts	[9]
MACS	Sliding window	Indirect	Local Poisson	P-value	P-value	[8]
QuEST	Gaussian kernel density	Indirect	Poisson	Height threshold	Q-value	[13]
SISSRs	Sliding window	Direct		Tag counts with sign change	P-value	[12]
NEXT-peak	NEXT-peak kernel density	Direct	Poisson per base pair	Likelihood	Estimated binding intensity (2ν)	

## Results

### Fitting the NEXT-peak model to ChIP-seq data

Using ChIP-seq datasets for three TFs: STAT1 [15], NRSF, and ZNF143 (see Methods for details), results with and without mappability information were examined; for each dataset, only the better of the two are presented here. Mappability information improved results for STAT1, but degraded results for NRSF and ZNF143.

**Figure 1:**
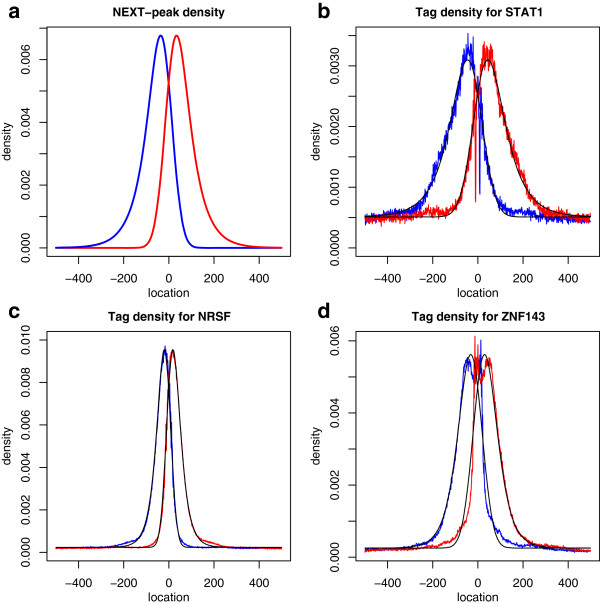
**Profile of the normal-exponential two-peak (NEXT-peak) density.** (**a**) An example of NEXT-peak density profile without fitting to a particular dataset. The blue curve is for a tag profile on the left (positive) strand, the red curve is for the right (negative) strand. Parameter values are *β* = 60, and *σ* = 40 (see Methods). The two density curves mirror each other around the center location. (**b**) Tag profile of STAT1 ChIP-seq data. From the motif search, thousands of motif sites were found. The cumulative tag counts were rescaled and displayed as densities. (**c**) NRSF. (**d**) ZNF143. Table 2 reports estimated values $\hat{\beta }$ and $\hat{\sigma }$ for each dataset.

Searches with position-specific scoring matrices from JASPAR [16] yielded candidates for actual STAT1, NRSF, or ZNF143 sites within each region with a binding event. The searches used the p-value cut-off 5×10^-6^ for all datasets. See Methods for details on the p-value computation for finding motif sites. Figure 1a shows a density of the normal-exponential two-peak (NEXT-peak) model (see Methods). Figure 1b-d displays the tag number, normalized to a probability density, for each location around the position of the candidate sites. The observed tag density is superimposed on the estimated density (derived from model estimates of the expected tag numbers, $\lambda_{j}^{R}$ or $\lambda_{j}^{L}$ in the Methods). Maximum likelihood estimation on the NEXT-peak model produced parameter estimates underlying $\lambda_{j}^{R}$ and $\lambda_{j}^{L}$. Table 2 reports estimated parameter values for each dataset.

**Table 2:** Summary of ChIP-seq datasets

**Dataset**	**tag length**	**motif length**	**number of tags**	$\hat{\beta }$	$\hat{\sigma }$	**Genome**
STAT1	27	15	15.1 million	73.5	52.8	Human build 36.1
NRSF	36	21	33.1 million	30.4	23.6	Human build 36.1
ZNF143	36	20	25.2 million	35.3	21.6	Human build 36.1

For STAT1, the observed tag counts follow the density curve of the NEXT-peak model with a small difference in terms of average trend, except for two unexplained dips (Figure 1b). The dips (one for right tags, and one for left tags) display symmetry around the binding site. The tag counts for NRSF (Figure 1c) also follow the NEXT-peak density with a small trend difference. The tag counts for ZNF143 (Figure 1d) show a larger trend difference from the NEXT-peak density, perhaps because the ChIP experiment was noisier.

### Examples of regions with unmappable locations

**Figure 2:**
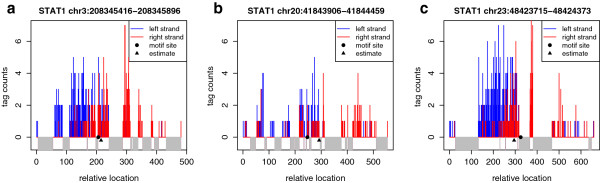
**A plot of regions with large number of unmappable locations from STAT1 ChIP-seq.** Tag counts in the left strand are shown as blue bars, tag counts in the right strand, as red bars. The unmappable locations are marked by grey blocks. The circles represent motif sites; the triangles, estimated sites. (**a**) 49% locations are unmappable. (**b**) 41% unmappable. (**c**) 38% unmappable.

Figure 2 shows three regions with large number of unmappable locations from STAT1 data. In Figure 2, unmappable locations are marked by grey blocks. In Figure 2a, 49% of locations are unmappable; in Figure 2b, 41%; and in Figure 2c, 38%. The circles indicate motif sites; the triangles, estimated sites from the NEXT-peak model. The estimated sites approximate the motif sites reasonably well. The estimated tag counts due to the binding event are 636.8, 264.3, and 699.3; the total observed tag counts in the region are 396, 209, and 492. Although tags at unmappable locations are not observable, the NEXT-peak model increases the corresponding estimated tag counts to compensate. The compensation permits NEXT-peak to sharpen estimates of binding strength.

### Comparison of the programs: top 2,000 peaks

To compare peak-calling programs, we run NEXT-peak along with other popular programs like HPeak [10], spp package (WTD and MTC) [11], CisGenome [9], MACS [8], QuEST [13], and SISSRs [12] (see Background for details). On running these programs including NEXT-peak, following standard practice, we used the default parameters to ensure reproducibility. As the first stage of comparison, we consider the 2,000 top peaks from each. The NEXT-peak program uses the estimated tags per binding event ($\hat{\nu }$) to rank its peaks. We considered every peak called within 250 bp of a candidate site (as determined by position-specific scoring matrix search) to be a true positive (TP). Our primary performance measure was the number of TPs within the 2,000 highest peaks. (Precision provides a standard but equivalent performance measure: the precision at 2,000 positives is the number of TPs within the 2,000 top peaks divided by the constant, 2,000.) Our secondary performance measures considered placement of TP peaks: (1) the mean distance between a TP peak center and the nearest motif site, and (2) the mean bias between a TP peak center and the nearest motif site. A TP peak upstream of the nearest motif site contributes to a negative bias; downstream, a positive bias. Thus, small distances and biases are desirable.

Table 3 contains summary statistics for various peak-calling programs. For STAT1, NEXT-peak found 781 TPs, a full 41 TPs more than any other program. For NRSF, NEXT-peak found 1,507 TPs, more than any other program; MTC was the second at 1,498 TPs. For ZNF143, NEXT-peak found 707 TPs, less than only MACS (709 TPs). For all three datasets, NEXT-peak had the smallest mean distances among all programs; it had one of the smallest biases as well. In addition, NEXT-peak is the only program that produced small biases for all three datasets. All other programs show a noticeable bias in at least one dataset. Specifically, HPeak and QuEST had a noticeable bias in STAT1; WTD, MTC, MACS and SISSRs had a noticeable bias in NRSF and ZNF143; CisGenome had a noticeable bias in STAT1 and ZNF143.

**Table 3:** Program result summary for ChIP-seq datasets

	**STAT1**			**NRSF**			**ZNF143**		
**Program**	**#motif sites**	**mean distance**	**mean bias**	**#motif sites**	**mean distance**	**mean bias**	**#motif sites**	**mean distance**	**mean bias**
HPeak	726	23.6	12.0	1489	7.5	0.2	698	26.0	0.5
WTD	732	20.2	0.7	1487	17.4	−16.5	698	26.1	−13.4
MTC	737	18.8	1.3	1498	16.7	−15.8	697	25.7	−12.2
CisGenome	716	28.5	11.3	1471	8.0	0.3	681	27.6	−15.1
MACS	740	24.4	−0.9	1491	18.5	−17.4	709	27.2	−13.8
QuEST	715	24.9	11.2	1105	8.3	−0.4	703	22.0	1.2
SISSRs	659	19.6	−0.2	1413	18.1	−16.8	661	29.1	−14.3
NEXT-peak	781	16.7	−1.6	1507	6.0	−0.5	707	20.3	0.6

### Comparison of the programs: top peaks in general

**Figure 3:**
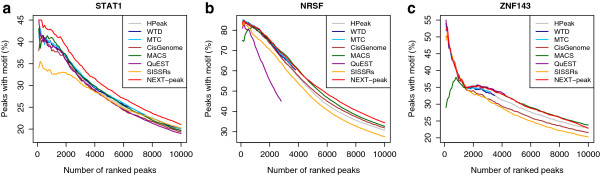
**A plot of percentage of top peaks with motif.** (**a**) STAT1. (**b**) NRSF. (**c**) ZNF143. Some curves were truncated, because QuEST called fewer than 3,000 peaks; and WTD and MTC, fewer than 4,000 peaks. (Large percentages are desirable.)

The previous section gives performance measures based on the top 2,000 peaks called by each program. Researchers might wish to compare the measures based on lists of top peaks with different truncations, e.g., lists truncated at rank 1,000, 4,000, or 10,000. Figure 3 shows the precision (fraction of TPs among the top peaks) for top peaks truncated at ranks up to 10,000. For each rank *r* in the x-axis, the precision is computed for cumulative peaks between rank 1 and rank *r*. As expected, precision generally decreased with the length of the list. For STAT1, NEXT-peak had the largest precision (of all programs examined) over the full range of lengths, up to 10,000 peaks. For NRSF, NEXT-peak had nearly the best precision up to 4,500 peaks (and in fact, it had the best precision at 2,000 peak; see the previous section.); NEXT-peak had the best precision between 4,500 and 10,000 peaks. For ZNF143, NEXT-peak had near the best precision up and 10,000 peaks. For ZNF143, MACS performed similarly to NEXT-peak between 1,500 and 10,000 peaks, but MACS had a significantly poorer performance between 0 and 1,500 peaks compared to other programs.

**Figure 4:**
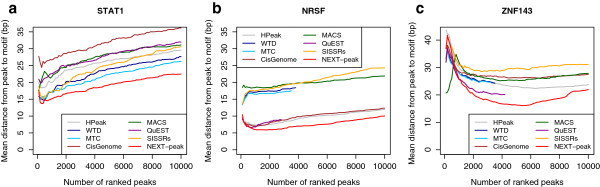
**A plot of mean distance between top peaks and motif.** (**a**) STAT1. (**b**) NRSF. (**c**) ZNF143. Mean distances are average distances between motif sites and estimated sties, where estimated sites contain a motif site within 250 bp distance. (Small distances are desirable.)

Figure 4 shows mean distances for TP peaks ranked up to 10,000. In all three datasets, for the most of the range up to rank 10,000, NEXT-peak had the smallest mean distances. Note that other programs did not show the same consistency among three datasets in terms of mean distances. For example, MTC was the second best in STAT1 but performed poorly for NRSF; QuEST was the second best in ZNF143 but performed poorly for STAT1.

**Figure 5:**
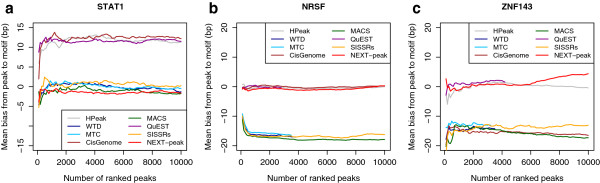
**A plot of mean bias between top peaks and motif.** (**a**) STAT1. (**b**) NRSF. (**c**) ZNF143.The bias is the (signed) distance in bp between an estimated site and the nearest motif site. (Small biases are desirable.) For all datasets, NEXT-peak biases were near zero. NEXT-peak was the only program with near-zero bias for all three datasets.

Figure 5 shows mean biases up to 10,000 peaks. As noted in the previous section, NEXT-peak is the only program showing small biases for all three datasets. Any other program shows a noticeable bias in at least one dataset. That is, for ZNF143, only NEXT-peak, QuEST, and HPeak had small biases, but QuEST and HPeak had noticeable biases in STAT1, making their performances highly dependent on the dataset at hand.

### Correlation of estimated standard deviation and distance to motif site

**Figure 6:**
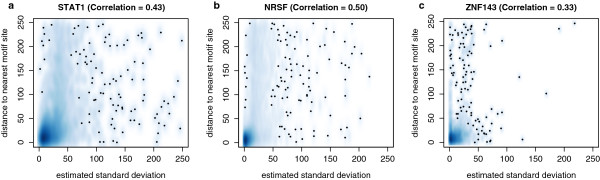
**Smooth scatterplots for estimated standard deviation vs. actual distance to nearest motif site.** (**a**) STAT1. (**b**) NRSF. (**c**) ZNF143. Both axes truncated values at 250 bp. For all datasets, the estimated standard deviation and the actual distance to the nearest motif site had a positive Pearson correlation coefficient. Dark regions represent high densities of data points and small dots represent isolated points. The majority of data points are located at the bottom-left corner for all three panels, hence most standard deviation estimates are small and actual distances to the motif site are also small in general.

Unlike other programs, NEXT-peak indicates the accuracy of a peak’s estimated location by estimating the corresponding standard deviation. Figure 6 displays smooth scatterplots for the estimated standard deviation versus distance to the nearest candidate site. (The plot is truncated on both axes at 250 bp, to dampen the influence of outliers caused by the omission of a true site among the candidate sites.) Dark regions represent high densities of data points and small dots represent isolated points. Ideally, all points should fall near the line y= x. Most data points, however, are concentrated at the bottom-left corner for all three plots. That is, most standard deviation estimates are small and actual distances to the motif site tend to be small as well. The Pearson correlation coefficients corresponding to the smooth scatterplots are 0.43 (STAT1), 0.50 (NRSF), and 0.33 (ZNF143), indicating that although the relationship is rather weak, the estimated error is positively correlated with the actual distance between the estimated peak location and the motif site.

## Discussion

Alone among existing peak-calling programs, NEXT-peak analyzes data with a parametric statistical model. Of the existing programs, therefore, it alone provides a principled foundation for elaborating the statistical analyses of ChIP-seq data. One obvious elaboration is to model multiple binding events in a region. This work is currently underway and the results will be reported elsewhere.

NEXT-peak can estimate the average fragment length, even if the experiment does not measure the average fragment length. Let *d* denote the tag length, e.g., *d*=27 for STAT1. In the NEXT-peak model, the average distance from a fragment end to a cross-link is *β*. The average distance between the fragment ends is therefore 2*β* + *d* − 1 (because the “location” of the right end is the leftmost position of the corresponding tag). For the STAT1 dataset, $\hat{\beta }$ =73.5 and *d* =27, so the NEXT-peak model estimate of the average fragment length is 173.0, consistent with a previous estimate of 174 [15]. For NRSF data, $\hat{\beta }$ =30.4 and *d*=36, the estimate of the fragment length is 95.8, and for GABP data, $\hat{\beta }$ =35.3 and *d*=36, the estimate is 105.6.

Existing programs simply discard ambiguously mapped reads. In contrast, NEXT-peak explicitly models the locations where reads do not map uniquely into the reference genome. NEXT-peak can therefore adjust for ambiguous mapping while estimating the total number of tags in a region, thereby sharpening its estimates of TF binding strength. Sharper estimates of binding strength can promote better physical interpretation of ChIP-seq results.

Existing peak-calling programs require tedious visual screening of up to tens of thousands of binding regions, to eliminate experimental artifacts like spikes in tag numbers caused by PCR anomalies. The goodness-of-fit tests in NEXT-peak can reduce the burden of visual screening. Moreover, the same tests can detect the presence of multiple TF binding sites, which are usually found in regions longer than those containing PCR anomalies. The long regions with small p-values can therefore be set aside for further, more intensive analyses, such as searching for multiple binding events or sequence motifs.

For STAT1, the observed tag distribution follows the NEXT-peak density closely, indicating that the NEXT-peak model captured the essence of the physical processes in the ChIP-seq experiment. Consequently, NEXT-peak outperformed its competitors, possibly because the NEXT-peak model successfully mimicked the true experimental kernel. On the other hand, for ZNF143, the observed tag distribution is somewhat deviated from the NEXT-peak density, possibly degrading NEXT-peak’s performance slightly. The observed tag density might reflect a mixture of multiple binding events, however, resulting from TF binding fluctuating between different protein complexes. Mass and structural differences between the protein complexes could cause binding locations or mean fragment lengths to fluctuate. Conventional motif analysis or a more elaborate model including multiple binding sites might expose the protein-protein interactions, however.

By adding mappability information, STAT1 increased true-positive binding sites by 4.0% on average. Unlike STAT1, mappability information for NRSF and ZNF143 actually degraded the performance of NEXT-peak: on average, it decreased true-positives by 0.5% and 0.6%, a surprising result given that both NRSF and ZNF143 had large numbers of mapped tags (33.1 millions and 25.2 millions). The truncated read length for NRSF and ZNF143 was 36, however, much larger than read length of 27 for STAT1. Thus, fewer genomic locations were mapped ambiguously for NRSF or ZNF143 (10.3%) than for STAT1 (16.2%), diminishing NEXT-peak’s ability to enhance its performance by adding mappability information.

This article examined three ChIP-seq datasets with a single dominant binding motif, permitting motif sites to serve as surrogates for the true binding sites. In general, however, even with an antibody specific to a protein, protein-protein interactions between TF molecules might cause multiple TFs (and hence, multiple motifs) to cross-link to an antibody. The two global parameters *σ* (the standard deviation for the cross-link locations) and *β* (the intensity of the Poisson process modeling shearing) then require delicate estimation. One could select a few hundred of the most tag-rich regions. One could screen the regions visually, choosing the ones with a good fit to the dual normal-exponential density and then estimate *σ* and *β*. Alternatively, one could perform a motif search on the tag-rich regions. The observed tag density for each motif then can be fit to the NEXT-peak model. Thus, NEXT-peak can analyze any ChIP-seq experiment, even without specific information on the protein interactions.

## Conclusions

We proposed a new statistical model for identifying binding sites from ChIP-seq data. The model successfully mimics the underlying data-generating process in ChIP-seq experiments by using the dual density of a normal-exponential two-peak model. The NEXT-peak program produced better prediction with more true positives and a better spatial resolution than any other program tested. The NEXT-peak program tests the validity of its underlying NEXT-peak model without depending (as many programs do) on an unrealistic assumption of a global uniform background tag distribution. The NEXT-peak program stands alone in quantifying errors by reporting a standard error for its estimates of binding intensity. Moreover, smooth scatterplots showed that its standard errors are informative about errors in motif location, as estimated from external standards. The NEXT-peak program also provides a goodness-of-fit test, automating screening of the spurious binding, and work is in progress to extend its model to locate multiple binding events in a region.

## Methods

### ChIP-seq datasets

Our analysis used ChIP-seq datasets corresponding to three TFs: STAT1, NRSF, and ZNF143. Because the three datasets correspond to known binding motifs, they provide a gold-standard for evaluating peak-calling programs [2]. Table 2 presents summary statistics for the three datasets.

The STAT1 dataset [15] was downloaded at http://www.bcgsc.ca/downloads/chiptf/human/STAT1/stimulated/july_23_2008/. The NRSF[SRA:SRR577995] and ZNF143[SRA:SRR243553] datasets were downloaded from the SRA database at http://www.ncbi.nlm.nih.gov/sra. Bowtie [17] mapped tags into a reference human genome (NCBI Build 36.1) for all three datasets. Mismatches of up to 2 bases were permitted, if they mapped uniquely within the genome.

For all datasets, the PeakSeq suite [5] then determined whether tag sequences in the reference genome were unique. PeakSeq requires tags of a uniform length. The tags for the downloaded STAT1 dataset, however, had varying length although most tags had length 27. We truncated the tags to length 27, if they were longer, or we discarded them, if they were shorter. Thus, we used the mappability information for 27 bp tags to approximate the complete STAT1 data. The downloaded NRSF had tags of length 50 and the downloaded ZNF143 had tags of length 40. We truncated tags from both datasets to length 36 to make it easy to investigate the effect of the mappability information (36 bp mappability information was used). Additional file 1 also reports on results from three additional datasets, MAX, GABP, and FoxA1.

### Notations for the ChIP-seq data

Let some preliminary method (see NEXT-peak algorithm for detail) flag possible cross-links in candidate genomic regions *R*_*r*_ (*r* = 1, …, *S*). Computational time is a consideration, because *S* can be on order of 10^4^ or more. Consider a specific genomic region *R*_*s*_, where *s* ∈ {1, …, *S*}, and let *R*_*s*_ have width *w*_*s*_, with the nucleotide bases having coordinates 1, …, *w*_*s*_. Call the forward and backward DNA strands “left” and “right”, so the bases at each location *j* on the left and right strands are complementary (*j* = 1, …, *w*_*s*_). Let $X^{0}$ denote the set of locations within *R*_*s*_ where a tag sequence is not unique within the genome, so the corresponding tag maps ambiguously.

The superscripts “*L*” and “*R*” distinguish quantities pertaining to the left and right strands: note in particular that “*L*” and “*R*” are not exponents. Within *R*_*s*_, let a total of *n*^*L*^ “left tags” be observed on the left strand; *n*^*R*^ “right tags”, on the right strand. Define the “location” of a tag as its leftmost position. Let the location of the left tags map to $x_{i}^{L}\in \left\{1,\ldots ,w_{s}\right\}$ (*i* = 1, …, *n*^*L*^); the location of the right tags, to $x_{i}^{R}\in \left\{1,\ldots ,w_{s}\right\}$ (*i* = 1, …, *n*^*R*^). Thus, the data are $X=\left(x_{1}^{L},\ldots ,x_{n^{L}}^{L},x_{1}^{R},\ldots ,x_{n^{R}}^{R},X^{0}\right)$ where no location $x_{i}^{L}$ or $x_{i}^{R}$ is in $X^{0}$.

An alternative representation is occasionally useful. Let $y_{j}^{L}\in \left\{0,1,2,\ldots \right\}$ be the number of left tags observed at location *j* ∈ {1, …, *w*_*s*_}; $y_{j}^{R}$, the number of right tags observed at position *j*. Tags cannot be observed at locations $j\in X^{0}$. Thus, $\left(y_{1}^{L},\ldots ,y_{w}^{L},y_{1}^{R},\ldots ,y_{w}^{R},X^{0}\right)$ provides an equivalent representation of the data **X**. The model parameters for the data in *R*_*s*_ are (*μ*_*s*_, *ν*_*s*_, *ρ*_*s*_) and (*σ*^2^, *β*), defined below. Parameters specific to the region *R*_*s*_ are subscripted with “*s*”; the parameters common to all genomic regions lack subscripts.

### Dual density for a binding event

Let the standard normal distribution have the density function *ϕ*(•) and the cumulative distribution function *Φ*(•). Each observed right tag location $x_{i}^{R}$ in *R*_*s*_ corresponds to an underlying (and unobservable) random variate *ξ*_*i*_, the coordinate of the corresponding cross-link. For mathematical convenience, assume *ξ*_*i*_ ~ *N*(*μ*_*s*_, *σ*^2^), i.e., *ξ*_*i*_ is a normal variate with mean *μ*_*s*_ (specific to *R*_*s*_) and variance *σ*^2^ (common to *R*_*r*_ for *r* = 1, …, *S*). The density of the distribution of *ξ*_*i*_ is

$$\pi \left(\xi_{i}\left|\mu_{s},\sigma^{2}\right)\right)=\frac{1}{\sigma }\phi \left(\frac{\xi_{i}-\mu_{s}}{\sigma }\right).$$

Assume that upon shearing, the breaks in the DNA form a Poisson process over the entire genome. Thus, the break point at location $x_{i}^{R}$ ($x_{i}^{R}>\xi_{i}$) corresponds to an exponential random variate $x_{i}^{R}-\xi_{i}$. For simplicity, assume that the mean *β* of the exponential distribution is common to all regions *R*_*r*_ (*r* = 1, …, *S*), so the density function corresponding to $x_{i}^{R}$ is

$$\pi \left(x_{i}^{R}\left|\xi_{i},\beta \right)\right)=\frac{1}{\beta }\exp\left(-\frac{x_{i}^{R}-\xi_{i}}{\beta }\right)$$

for $x_{i}^{R}>\xi_{i}$, and 0 otherwise.

The joint density of$\left(x_{i}^{R},\xi_{i}\right)$is therefore$\pi \left(x_{i}^{R},\xi_{i}\left|\mu_{s},\sigma^{2},\beta \right)\right)=\pi \left(x_{i}^{R}\left|\xi_{i},\beta \right)\right)\cdot \pi \left(\xi_{i}\left|\mu_{s},\sigma^{2}\right)\right)$. Integrate *ξ*_*i*_ out, to derive the density function of $x_{i}^{R}$:

fRxiRμs,σ2,β=∫πxiR,ξiμs,σ2,βdξj=ΦxiR−μs+σ2β−1σ1βexp−1βxiR−μs+12σ2β−1

**Figure 7:**
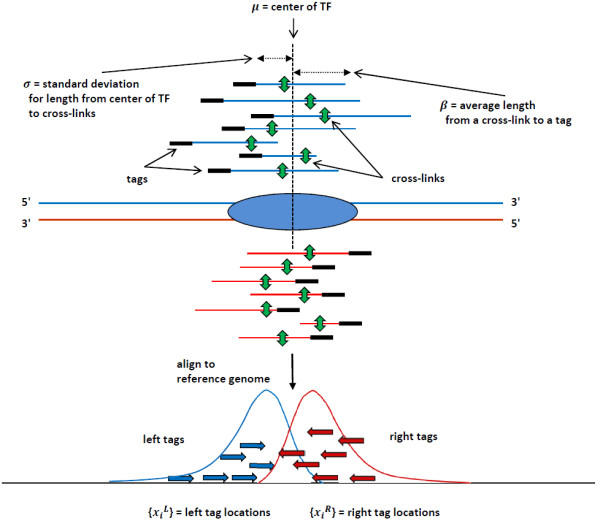
**ChIP-seq experiment with NEXT-peak model parameters.** A genomic location of the center of the TF is denoted as *μ*. Green bi-directional arrows represent cross-links between a TF and a genomic sequence. Cross-links are assumed to be normally distributed with standard deviation *σ*. Tags are shown as small black rectangles at the 5′ end of fragments. The distance from a cross-link to a tag location is assumed to be exponentially distributed with mean *β*. When tags are mapped to a reference genome, then tags are projected onto the corresponding genomic locations. Blue arrows represent “left” tags mapped on the forward strand; red arrows, “right” tags mapped on the backward strand. The tag distribution is the NEXT-peak under the previous assumptions.

The above distribution is a marginal distribution of the normal-exponential joint density, which we call a “normal-exponential” distribution in short. Figure 7 shows a schematic representation for the role of parameters *σ* and *β* in a ChIP-seq experiment. Figure 1a shows a normal-exponential density for both left and right tags (as discussed in Results).

### Poisson regression model for the observed tags

Within *R*_*s*_: (1) let *ν*_*s*_ be the expected number of right tags for each TF molecule that binds; (2) let *ρ*_*s*_ be the uniform background intensity of right tags; and (3) let $\lambda_{j}^{R}$ be the expected number of right tags at location *j*. Assume

$$\lambda_{j}^{R}=\nu_{s}f^{R}\left(j\left|\theta \right)\right)+\rho_{s}.$$

Approximate the sum over all locations *j* with an integration, to produce a consistency check: *ν*_*s*_ = ∫ *ν*_*s*_ · *f*^*R*^(*x*|**θ**)*dx*. The expected total number of right tags within *R*_*s*_ due to binding is therefore approximately *ν*_*s*_, *ν*_*s*_ = 0 being equivalent to the absence of TF binding in *R*_*s*_. On the other hand, the expected total number of right tags within *R*_*s*_ due to noise is *w*_*s*_*ρ*_*s*_.

Let $Y_{j}^{R}$ be the random variable that counts right tags at *j*, and assume *Y*_*j*_^*R*^ has a Poisson distribution with mean $\lambda_{j}^{R}$, i.e.,

$$\mathrm{Pr}\left(Y_{j}^{R}=y\right)=\exp\left(-\lambda_{j}^{R}\right)\frac{{\left(\lambda_{j}^{R}\right)}^{y}}{y!},$$

for *y* ϵ {0, 1, 2, …}.

The models for the tag locations $\left\{x_{j}^{L}\right\}$ and $\left\{x_{j}^{R}\right\}$ on the left and right strands share the parameters (*μ*_*s*_, *σ*^2^, *β*, *ν*_*s*_, *ρ*_*s*_) and differ only in mirroring the tag location densities around the link location *μ*_*s*_, i.e.,

$$f^{L}\left(\mu_{s}-z\left|\mu_{s},\sigma^{2},\beta \right)\right)=f^{R}\left(\mu_{s}+z\left|\mu_{s},\sigma^{2},\beta \right)\right).$$

The models for the left and right tags share *ν*_*s*_ and *ρ*_*s*_, so as in the model for the right tags, *ν*_*s*_ is the expected number of left tags for each TF molecule that binds, and *ρ*_*s*_ is the uniform background intensity of left tags.

Figure 1a shows a normal-exponential two-peak (NEXT-peak) density. In this example, *μ* = 0, *β* = 60, and *σ* = 40. The two density curves mirror each other around the center location *μ* = 0. The curves are asymmetrical distributions skewed in opposite directions. Although both tails of each density rapidly approach zero, one tail approaches zero much faster than the other. Figure 1a motivates the model name: the “normal-exponential two-peak” (NEXT-peak) model.

Let **θ**_*s*_ = (*μ*_*s*_, *ν*_*s*_, *ρ*_*s*_). Under the NEXT-peak model in Figure 1a, the likelihood of the data $\left(y_{1}^{L},\ldots ,y_{w}^{L},y_{1}^{R},\ldots ,y_{w}^{R},X^{0}\right)$ in *R*_*s*_ is

$$L\left(\sigma ,\beta ,\theta_{s}\right)=\prod_{j\notin X^{0}}\left[\mathrm{Pr}\left(Y_{j}^{R}=y_{j}^{R}\left|\sigma ,\beta ,\theta_{s}\right)\right)\cdot \mathrm{Pr}\left(Y_{j}^{L}=y_{j}^{L}\left|\sigma ,\beta ,\theta_{s}\right)\right)\right].$$

Thus, the likelihood of the complete dataset is

$$L\left(\sigma ,\beta ,\theta \right)=\prod_{r=1}^{S}L\left(\sigma ,\beta ,\theta_{s}\right)$$

where **θ** = (**θ**_*r*_ : *r* = 1, …, *S*).

Because *σ* and *β* do not depend on the region *R*_*s*_, training data can yield maximum likelihood estimates (MLEs) $\hat{\sigma }$ and $\hat{\beta }$. Fix the values $\sigma =\hat{\sigma }$ and $\beta =\hat{\beta }$, so the remaining parameters requiring estimation for the NEXT-peak model are **θ** = (**θ**_1_, …, **θ**_*S*_). Maximization of the profile likelihood $L\left(\theta_{s}\right)=L\left(\hat{\sigma },\hat{\beta },\mu_{s},\nu_{s},\rho_{s}\right)$ then yields the estimate ${\hat{\theta }}_{s}=\left({\hat{\mu }}_{s},{\hat{\nu }}_{s},{\hat{\rho }}_{s}\right)$ within each region *R*_*s*_. The estimate’s components are: (1) the estimated mean location ${\hat{\mu }}_{s}$ of a binding event, (2) the estimated mean total number ${\hat{\nu }}_{s}$ of right (or left) tags within *R*_*s*_ due to the binding event, and (3) the estimated uniform background intensity ${\hat{\rho }}_{s}$ of right (or left) tags within *R*_*s*_. As usual, the inverse of the Fisher Information matrix (i.e., the inverse of the negative expectation of the Hessian of the log-likelihood) estimates the asymptotic variance-covariance matrix for **θ**_*s*_.

Let ${\hat{\hat{\theta }}}_{s}=\left({\hat{\hat{\mu }}}_{s},0,{\hat{\hat{\rho }}}_{s}\right)$ denote the maximum likelihood estimate (MLE) of **θ**_*s*_ = (*μ*_*s*_, *ν*_*s*_, *ρ*_*s*_) under the restriction *ν*_*s*_ = 0. To test whether or not a binding event occurred in *R*_*s*_, under the null hypothesis *H*_0_:*ν*_*s*_ = 0, the likelihood ratio (LR) statistic

$$\lambda =-2\log\left[L\left({\hat{\hat{\theta }}}_{s}\right)/L\left({\hat{\theta }}_{s}\right)\right]$$

has an asymptotic chi-square distribution with 1 degree of freedom. Unlike the null hypotheses in many existing programs, the NEXT-peak model does not assume a globally uniform background intensity. Instead, its locally uniform background intensity is equivalent to assuming that the expected number of background tags per location varies slowly enough to be almost constant within each region *R*_*r*_ (*r* = 1, …, *S*).

### Goodness-of-fit test

The following can test whether observed tag data are consistent with the NEXT-peak model. PCR over-amplification and other experimental artifacts can cause spikes in the observed tag distribution. Likewise, multiple binding events in a region *R*_*r*_ cause deviations from the unimodal density of the model. Accordingly, consider Pr (**D**|*H*_0_), the probability of the data **D** under the null hypothesis *H*_0_ of a NEXT-peak model. Under *H*_0_, the counts $y_{j}^{R}$ of right tags at location *j* are generated with the Poisson intensity $\lambda_{j}^{R}$ given above, the counts of the left tags being generated with the mirror intensity $\lambda_{j}^{L}$. Consider also the probability Pr (**D**|*H*_1_) of the data under an alternative model *H*_1_ where the underlying intensity $\lambda_{j}^{R}$ is unrestricted. The LR statistic 2 log[Pr(**D**|*H*_1_)/Pr(**D**|*H*_0_)] for the models follows a *χ*^2^ distribution, with degrees of freedom equal to the difference of the number of parameters in *H*_0_ and *H*_1_. The LR test yields a p-value for each region *R*_*s*_, with a small p-value indicating a poor fit within *R*_*s*_ to the NEXT-peak model underlying *H*_0_.

### P-value computation for finding motif sites

From a position-specific score matrix, any segment receives a score by adding the corresponding columns scores from the position-specific score matrix. The probability of observing the score or higher is computed based on the null model that nucleotides (A, C, G, and T) can appear at random with equal probabilities. We used the Staden’s method [18] for the convolution computation of the score distribution, The principal of our p-value computation has been used for PSI-BLAST [19] and A-GLAM [20], among others, for their p-value computation, where p-values are used to compute E-values.

### Screening based on goodness-of-fit tests

Like many peak-calling programs, NEXT-peak masks locations having anomalously many tags, but it does so in a principled manner, with p-values based on goodness-of-fit tests. Long regions with small p-values suggest multiple binding sites, so for all datasets NEXT-peak masked regions less than a certain length long and with small p-values. When motif site locations are available, based on the area under the precision curve up to the top 10,000 peaks (e.g. Figure 3), the NEXT-peak program automatically reports a cut-off recommendation for each dataset. The Results section uses the length 400 and the p-value 10^-8^ as a cut-off for STAT1 and the length 300 and the p-value 10^-4^ as a cut-off for NRSF and the length 400 and the p-value 10^-6^ as a cut-off for ZNF143, all suggested by the NEXT-peak output.

### NEXT-peak algorithm

**Figure 8:**
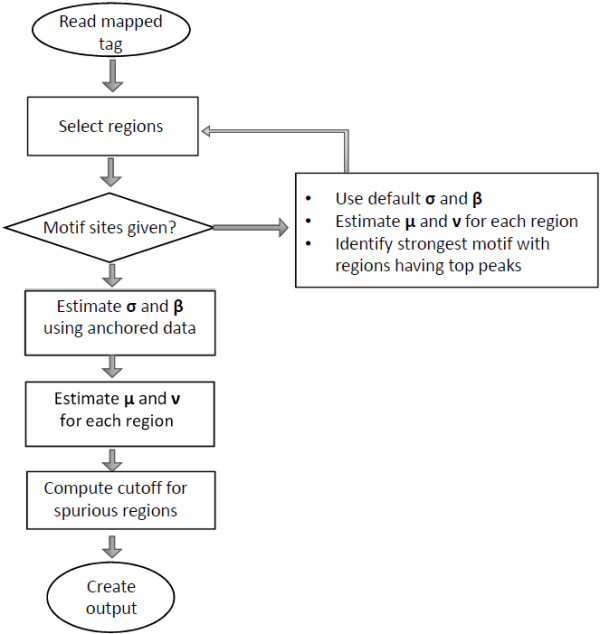
**A flowchart for NEXT-peak algorithm.** First, the program reads the mapped tags. Then, it selects regions based on the tag count with a user specified length of the window (default: 150) and a user specified minimum count (default: 15). If motif site locations are provided, it estimates *σ* and *β* using motif site locations. For an unknown motif, run the NEXT-peak program with default values (*σ* = 30 and *β* = 50), and identify the strongest motif from a motif search. For each region, the program estimates *μ* and *ν*. It computes the standard deviation estimates for these estimates. As a post-processing step, the program computes the region length and p-value cut-off recommendations to screen out potential spurious regions when the motif site information is available.

The NEXT-peak program goes through the following procedures for producing an output (Figure 8 shows a flowchart for the NEXT-peak program). (1) Read the mapped tag location file, e.g., from a Bowtie [17] output. (2) Select regions based on the tag count with a user specified length of the window (default: 150) and a user specified minimum count (default: 15). When a neighboring window has more than the minimum count, the window under scrutiny is combined with its neighbor. The region lengths range from the minimum length (default: 150) to several thousands. (3) When motif site locations are available, estimate *σ* and *β* by maximizing the likelihood using motif site locations. For a known TF, a publicly available motif pattern is used, e.g. from JASPAR [16]. For an unknown motif, run the NEXT-peak program with default values (*σ* = 30 and *β* = 50), and identify the strongest motif from a motif search. Alternatively, a user can estimate these parameters with selected regions. (4) For each region, estimate *μ* and *ν* by maximizing the likelihood. It computes the standard deviation estimates for these estimates. Then, perform a goodness-of-fit test for each region. (5) As a post-processing step, compute the region length and p-value cut-off recommendations to screen out potential spurious regions when the motif site locations are available.

### NEXT-peak software

The NEXT-peak program is implemented in C++. The code is publicly available at http://www.odu.edu/~nxkim/nextpeak/. A typical computation time is about 15 ~ 45 minutes, depending on the size of the input data.

## Competing interests

The authors declare that they have no competing interests.

## Authors’ contributions

NKK conceived the study, developed the model, developed the program, performed a comparison study, and drafted the manuscript. RVJ helped parameter estimation and performed a comparison study. JLS conceived the study, developed the model and drafted the manuscript. All authors read and approved the final manuscript.

## Supplementary Material

Additional file 1 **Supplementary material.** Supplementary material contains results for additional datasets, MAX, GABP, and FoxA1. This file contains four supplementary figures and two supplementary tables: **Figure S1.** A plot of percentage of top peaks with motif. Table S1 reports estimated values $\hat{\beta }$ and $\hat{\sigma }$ for each dataset. **Figure S2.** A plot of percentage of top peaks with motif. Some curves were truncated in (**a**), because QuEST called fewer than 5,000 peaks; MTC, fewer than 7,000 peaks; and WTD, fewer than 9,000 peaks. In (**b**), QuEST, WTD, and MTC called fewer than 4,000 peaks. **Figure S3.** A plot for mean distance between top peaks and motif. Mean distances are average distances between motif sites and estimated sites, where estimated sites contain a motif site within 250 bp distance. **Figure S4.** A plot of mean bias between top peaks and motif. The bias is the (signed) distance in bp between an estimated site and the nearest motif site. **Table S1.** Summary of additional ChIP-seq datasets. Basic characteristics of additional datasets. **Table S2.** Program result summary for additional ChIP-seq datasets. Click here for file
