# Supplementary material for: NEXT-peak: a normal-exponential two-peak model for peak-calling in ChIP-seq data
Source: BMC Genomics. 2013 May 25;14:349. doi: 10.1186/1471-2164-14-349 (PMC3672025; doi:10.1186/1471-2164-14-349)
Supplement: Additional file 1 — Supplementary material. Supplementary material contains results for additional datasets, MAX, GABP, and FoxA1. This file contains four supplementary figures and two supplementary tables: Figure S1. A plot of percentage of top peaks with motif. Table S1 reports estimated values β^ and σ^ for each dataset. Figure S2. A plot of percentage of top peaks with motif. Some curves were truncated in (a), because QuEST called fewer than 5,000 peaks; MTC, fewer than 7,000 peaks; and WTD, fewer than 9,000 peaks. In (b), QuEST, WTD, and MTC called fewer than 4,000 peaks. Figure S3. A plot for mean distance between top peaks and motif. Mean distances are average distances between motif sites and estimated sites, where estimated sites contain a motif site within 250 bp distance. Figure S4. A plot of mean bias between top peaks and motif. The bias is the (signed) distance in bp between an estimated site and the nearest motif site. Table S1. Summary of additional ChIP-seq datasets. Basic characteristics of additional datasets. Table S2. Program result summary for additional ChIP-seq datasets. [file 1471-2164-14-349-S1.pdf]

Supplementary material

NEXT-peak: a normal-exponential two-peak model for peak-calling in ChIP-seq data

Nak-Kyeong Kim, Rasika V. Jayatillake, and John L. Spouge

In addition to three datasets in the main article, three additional datasets were analyzed to compare performances of NEXT-peak and other programs. The MAX [SRA:SRR307020], GABP[SRA:SRR332363], and FoxA1[SRA: ERR022036] datasets were downloaded from the SRA database at http://www.ncbi.nlm.nih.gov/sra. Bowtie mapped tags into a reference human genome (NCBI Build 36.1) for all three datasets. Mismatches of up to 2 bases were permitted, if they mapped uniquely within the genome. The downloaded MAX had tags of length 34, the downloaded GABP had tags of length 37, and the downloaded FoxA1 had tags of length 36. We truncated tags from GABP to length 36 to investigate the mappability effect on the NEXT-peak. For a tag length 34 as in MAX, 12.0% of genomic locations were mapped ambiguously. For a tag length 36 as in GABP and FoxA1, 10.3% locations were mapped ambiguously, which are the same for NRSF and ZNF143.

To create a profile of the tag distribution around the binding location, position-specific scoring matrices from JASPAR (MAX and FoxA1) and TRANSFAC (GABP) were used to search for candidate bind sites. The searches used the p-value cut-off $5x10^{-6}$ for MAX and the p-value cut-off 10^{-6} for GABP and FoxA1. Figure S1 displays observed tag counts around the candidate sites along with a superimposed density of the normal-exponential two-peak (NEXT-peak) model. All three datasets show a reasonably good fit to the NEXT-peak model, although not ideal. Table S1 reports estimated parameter values for each dataset.

For the screening of the spurious binding, NEXT-peak automatically reports the recommended cut-offs. It reported the length 500 and the p-value 10^{-6} as a cut-off for MAX and the length 400 and the p-value 10^{-8} as a cut-off for GABP and the length 300 and the p-value 10^{-6} as a cut-off for FoxA1. Mappability information improves results for FoxA1, but not for MAX and GABP. We report better one.

Table S2 reports statistics when considering top 2,000 peaks from each predicted peaks from various peak-calling programs. For MAX, NEXT-peak found 776 TPs, second only to QuEST (779 TPs). For GABP, NEXT-peak found 570 TPs, surpassed only by MACS (575 TPs) and QuEST (586 TPs). For FoxA1, NEXT-peak found 560 TPs, surpassed only by MACS (563 TPs) and CisGenome (561 TPs). In terms of mean distances, NEXT-peak had the smallest at 19.9 in MAX. For both GABP and FoxA1, NEXT-peak had the

second smallest. In terms of mean bias, NEXT-peak had the smallest in MAX, For GABP and FoxA1, NEXT-peak was among a group of smaller biases.

Figure S2 shows a plot of percentage of top peaks with a motif site. For MAX, NEXT-peak had largest TPs between 3,000 and 10,000 peaks. Between 0 to 2,000 peaks, QuEST had the largest. Note that QuEST called fewer than 5,000 peaks for this dataset. For GABP, NEXT-peak produced near the largest TPs throughout the range. For FoxA1, TPs from all programs are close to each other. The NEXT-peak produced near the largest TPs in most range.

Figure S3 shows mean distances between peaks and the nearest motif sites. For MAX, NEXT-peak had smallest distances for almost all range. For GABP, NEXT-peak was among the smallest up to 5,000 peaks, and then mean distances increased as the peak number increased. For FoxA1, NEXT-peak had mean distances near the smallest.

Figure S4 shows mean bias from peaks to the nearest motif site. Along with HPeak and QuEST, NEXT-peak produced smaller biases for all three datasets, MAX, GABP, and FoxA1. WTD, MTC, MACS, and SISSRs produced noticeable biases for all three datasets. GisGenome produced a noticeable bias for MAX.

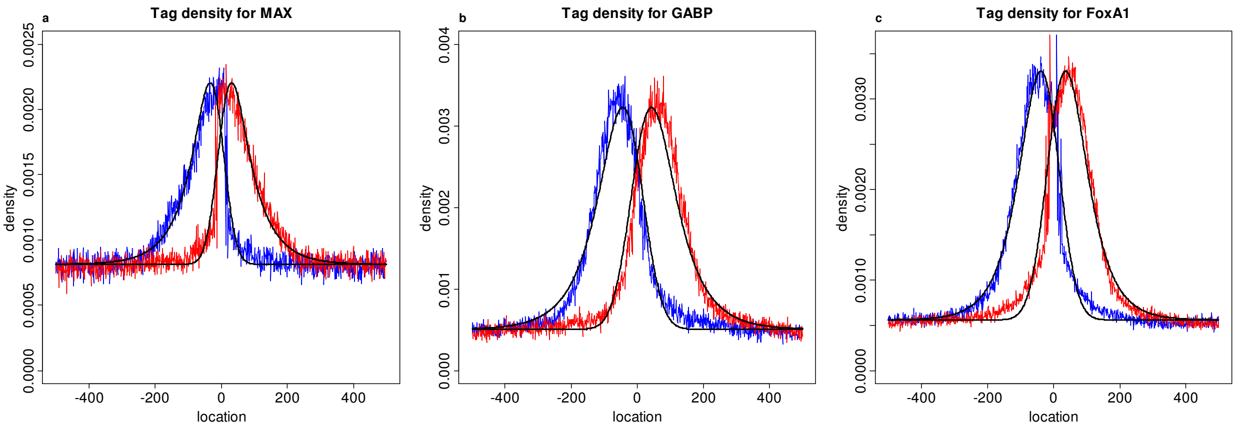

Figure S1 - A plot of percentage of top peaks with motif (a) MAX. (b) GABP. (c) FoxA1. This figure is analogous to Figure 2. Table S1 reports estimated values $\hat{\beta}$ and $\hat{\sigma}$ for each dataset.

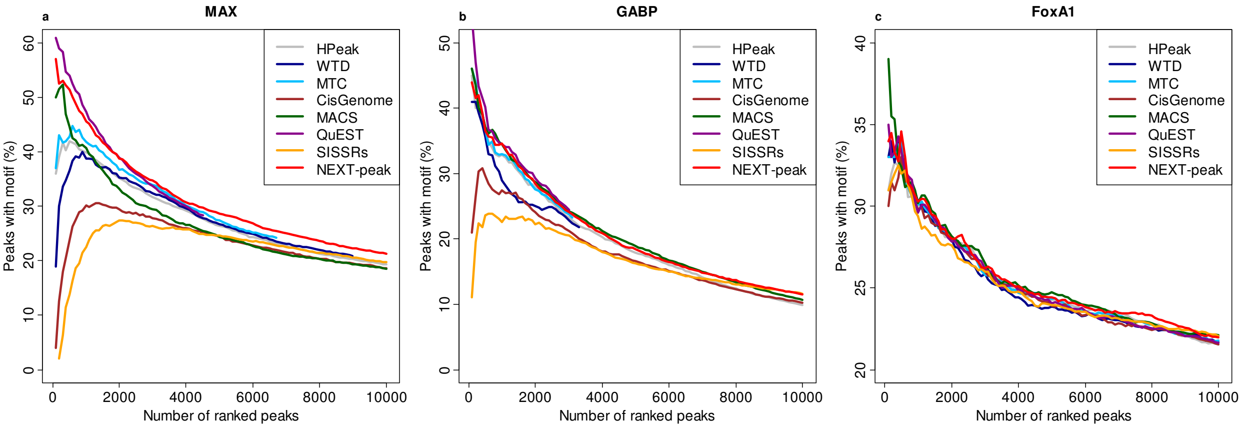

Figure S2 - A plot of percentage of top peaks with motif (a) MAX. (b) GABP. (c) FoxA1. This figure is analogous to Figure 3. Some curves were truncated in (a), because QuEST called fewer than 5,000 peaks; MTC, fewer than 7,000 peaks; and WTD, fewer than 9,000 peaks. In (b), QuEST, WTD, and MTC called fewer than 4,000 peaks.

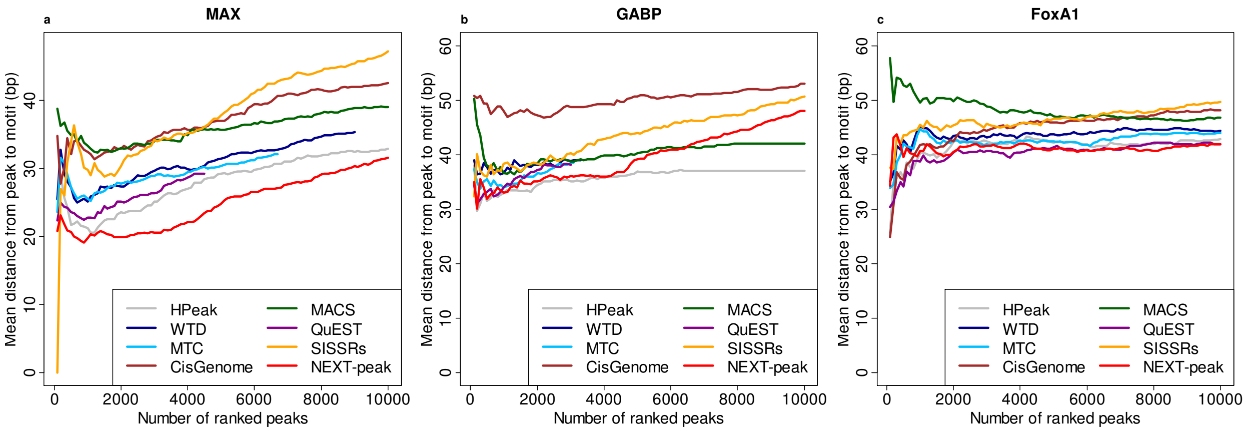

Figure S3 - A plot for mean distance between top peaks and motif (a) MAX. (b) GABP. (c) FoxA1. This figure is analogous to Figure 4. Mean distances are average distances between motif sites and estimated sties, where estimated sites contain a motif site within 250 bp distance. (Small distances are desirable.)

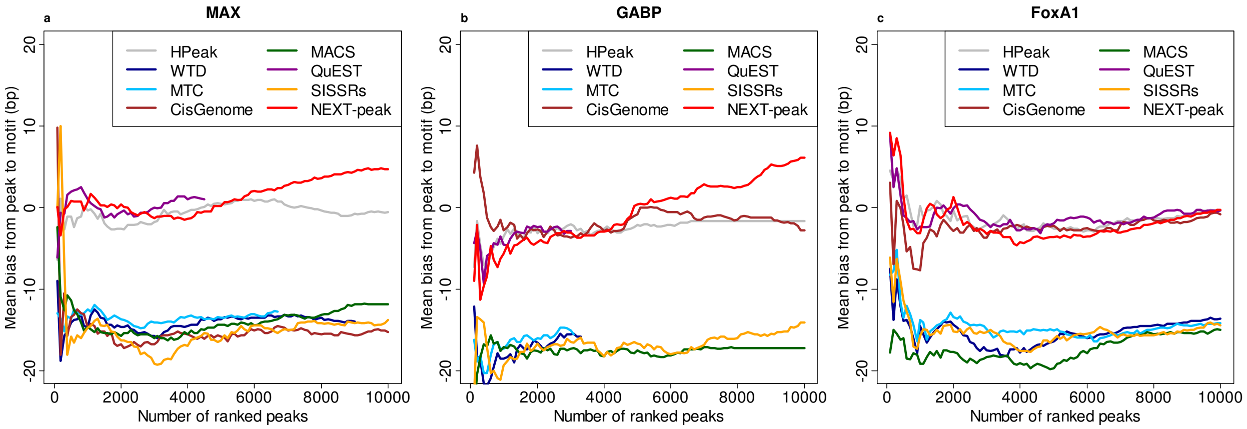

Figure S4 - A plot of mean bias between top peaks and motif (a) MAX. (b) GABP. (c) FoxA1. This figure is analogous to Figure 5. The bias is the (signed) distance in bp between an estimated site and the nearest motif site. (Small biases are desirable.)

Table S1 - Summary of additional ChIP-seq datasets

Dataset	tag length	motif length	number of tags	$\hat{\beta}$	$\hat{\sigma}$	Genome
MAX	34	10	15.0 million	63.3	31.8	Human Build 36.1
GABP	36	12	26.6 million	73.4	49.7	Human Build 36.1
FoxA1	36	11	22.9 million	59.3	46.6	Human Build 36.1

Table S2 - Program result summary for additional ChIP-seq datasets

Program	MAX			GABP			FoxA1		
	#motif sites	mean distance	mean bias	#motif sites	mean distance	mean bias	#motif sites	mean distance	mean bias
HPeak	704	23.5	-2.6	557	34.3	-3.2	559	42.3	-0.4
WTD	706	27.3	-14.7	501	38.1	-16.6	559	43.5	-14.4
MTC	733	27.8	-13.7	551	36.6	-16.1	557	42.6	-13.4
CisGenome	589	32.9	-16.8	482	47.2	-2.7	561	42.5	-2.9
MACS	664	33.0	-15.8	575	38.4	-17.6	563	49.9	-17.7
QuEST	779	25.1	-0.6	586	37.2	-2.4	557	40.3	-0.1
SISSRs	547	30.4	-16.4	445	38.4	-16.7	552	45.4	-15.2
NEXT-peak	776	19.9	0.3	570	35.0	-4.3	560	40.5	1.2
